# Mechanistic Interaction Study of Bromo-Noscapine with Bovine Serum Albumin employing Spectroscopic and Chemoinformatics Approaches

**DOI:** 10.1038/s41598-018-35384-6

**Published:** 2018-11-16

**Authors:** Damini Sood, Neeraj Kumar, Garima Rathee, Anju Singh, Vartika Tomar, Ramesh Chandra

**Affiliations:** 1 Drug Discovery & Development Laboratory, Department of Chemistry, University of Delhi, Delhi, 110007 India; 2 Nucleic Acids Research Laboratory, Department of Chemistry, University of Delhi, Delhi, 110007 India; 3 Dr. B. R. Ambedkar Centre for Biomedical Research, University of Delhi, Delhi, 110007 India

## Abstract

Bromo-Noscapine (BrNs) is a tubulin-binding cytotoxic agent with significant activity against breast and lung cancer. The mechanistic interaction insight into the binding of bovine serum albumin (BSA) with BrNs can provide critical information about the pharmacodynamics and pharmacokinetics properties. Here, various spectroscopic techniques and computational methods were employed to understand the dynamics of BrNs and BSA interaction. The intrinsic fluorescence of BSA was quenched by BrNs through a static quenching procedure. The stoichiometry of BrNs-BSA complex was 1:1 and binding constant of the complex was in the order of 10^3^ M^−1^ at 298 K. Based on thermodynamic analysis, it was deduced that binding process of the BrNs with BSA was spontaneous and exothermic, and the major forces between BrNs and BSA were van der waals forces and hydrogen bonding. Moreover, results of FT-IR, CD, UV spectra concluded significant conformational change in BSA on binding with BrNs. The *in vitro* findings were further confirmed by *in silico* assays. Molecular docking showed strong interactions with score of −8.08 kcal/mol. Molecular dynamics simulation analysis also suggested the stable binding with lower deviation in RMSD and RMSF values through persistent long simulation run. This study suggests optimal efficiency of diffusion of the BrNs into the bloodstream for the treatment of cancer.

## Introduction

The protein contents of body fluids are considered to be a vital index for the clinical diagnosis of any drug. Bioavailability of the drug under testing is important for its direct regulation, interaction and involvement in immunity generation and metabolism. Blood proteins are the main factors for the transportation and homing of drugs to the target molecules^1–3^. Mechanistic Interaction insights studies of the drug help in determining the factors that influence the protein conformational changes, protein folding, and ligand binding activity elucidation^4,5^. Over last decades, interaction studies of drugs with serum albumin proteins have attracted great interest to reach a step closer to preclinical trials.

Among the different blood proteins, human serum albumin (HSA) and bovine serum albumin (BSA) are the most abundant proteins with their indispensable role in drug transportation^6,7^. Bovine serum albumin is commonly used as a model protein for Human serum albumin^8–11^ due to its strong structural similarity, low procurement cost, and ease in availability^12,13^. The resemblance between BSA and HSA is 86% with respect to amino acid sequences and 75.6% in terms of identity^14^. In this regard, we have performed the mechanistic interaction study of BSA with target drug BrNs. BSA protein is of size 583 amino acids and made up of three linearly arrangement sub-domains which are also structurally homologous. BSA has two tryptophan residues which consisted of intrinsic domains^15^. Exploration of pharmacokinetics properties including the distribution, transportation, and excretion of the lead drug-ligand with BSA protein receptor and further assessment of molecular interactions are requisite studies prior to preclinical trials of lead drugs^16–20^.

**Figure 1:**
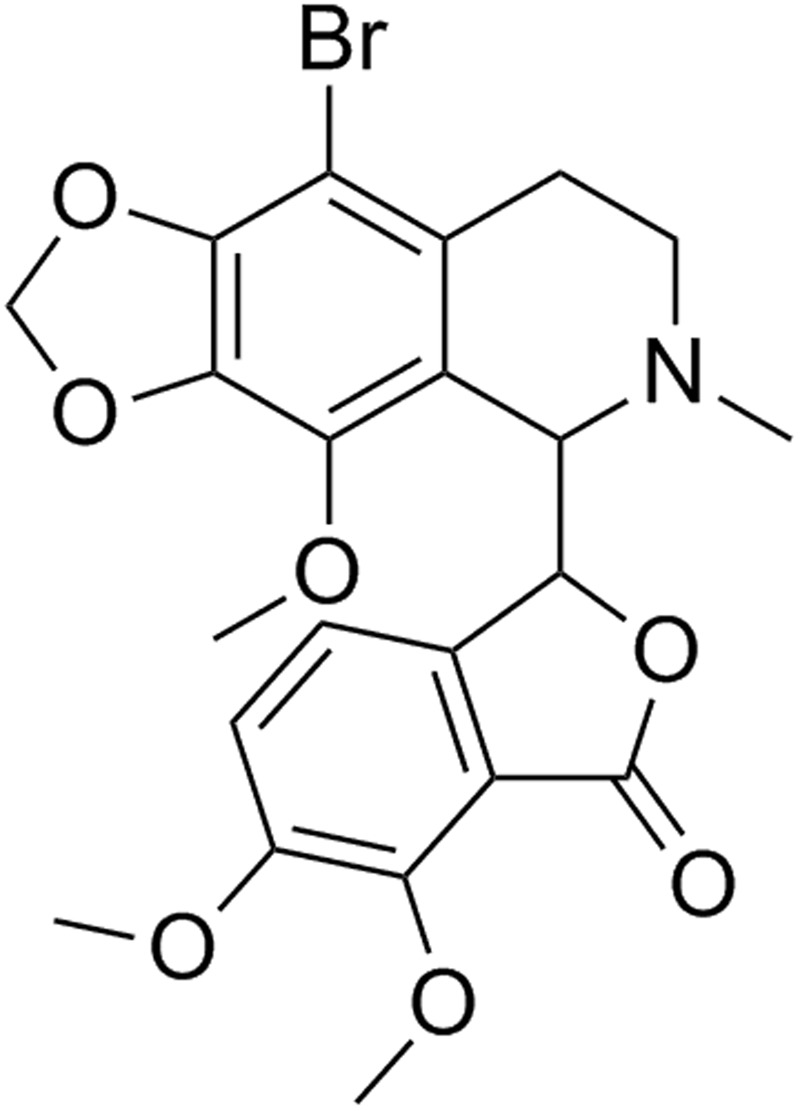
Chemical structure of Bromo-noscapine.

In this study, BrNs has been explored for its mechanistic interaction with BSA. BrNs is one of the potent analogues of noscapine scaffold with its higher anticancer activity. Noscapine is the phthalideisoquinoline alkaloid, which was first isolated from opium poppy^21^. Noscapine class are non narcotic, non addictive compounds and reported to posses the anticancer activity with its role to block the overexpression of tubulin protein^22,23^. BrNs (Fig. 1) is one of the efficient drugs of all noscapine analogues and belongs to the first generation noscapinoids, derived by chemical modification of isoquinoline and benzofuranone group of noscapine scaffold^24^.

BrNs has been reported to possess the significant anticancer activity against the non small cell lung cancerous cells, by its role in alteration in the tubulin polymerization during the hyper-regulation of cell cycle^25,26^. In one of the other reports, BrNs has been reported to possess the anti-inflammatory activity in the *in vitro* models mimicking the innate immune pathways^27^. Hence, herein interaction studies have been performed for BrNs interaction study with BSA protein, employing the spectroscopic analyses along with the computational assays. Computational assays involving the molecular docking and molecular dynamics simulation have been widely used to study ligand binding mechanism to the particular target proteins^28^. Molecular docking studies give insight to the binding conformations of ligand to the target molecule with determining the involved molecular interactions (mainly hydrophobic and hydrogen bonds). In addition, molecular dynamics simulation studies provide the depth knowledge of interactions at the atomic level of protein and help in studying the stability of interacting complex of ligand drug with target protein and conformational changes through root mean square deviation (RMSD) and root mean square fluctuation (RMSF) analysis. These parameters including the stability and flexibility of drug conformations are the potential factors to assess the functional and biological activity of the drug under investigation. Previously in many reports molecular docking and simulation studies have been performed to elucidate the mechanistic interaction of the ligand and also to design the potential anti-cancer drugs on the basis of binding of compound to binding groove of oncotarget proteins^29,30^. In the present work, spectroscopic analyses including fluorescence, FT-IR (Fourier-transform infrared) spectroscopy, ultra violet spectrophotometry (UV) and circular dichroism (CD) analysis have been employed to explore the interaction of BrNs with BSA in the simulative physiological conditions. Thereafter, molecular docking and simulation studies have been performed of BrNs with BSA.

## Results and Discussion

### Fluorescence Quenching of BSA by BrNs

**Figure 2:**
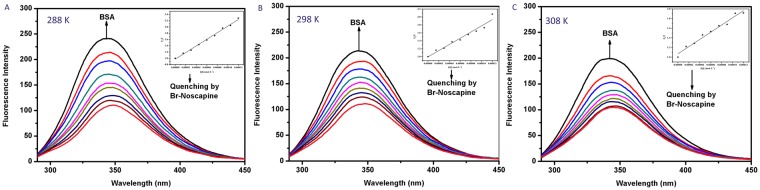
Fluorescence quenching spectra of BSA with BrNs at quenching spectra of BSA in the presence of BrNs at (**A**) 288 K (**B**) 298 K (**C**) 308 K. [BSA] = 15 μM, [BrNs] = 0 to 120 μM. Insets: Stern–Volmer plots of BSA-BrNs complex.

The unique ability of proteins to display intrinsic fluorescence has provided a pathway to understanding the changes in its environment upon quencher interaction^31,32^. The three natural amino acids- tryptophan (Trp), tyrosine (Tyr) and phenylalanine (Phe) are fluorescent, but Trp has the highest fluorescence quantum yield^33^. The quenching mechanism of the BSA-BrNs system was explored by analysing the fluorescence data. The gradual addition of BrNs caused a concomitant decrease in the fluorescence emission spectra of BSA at 344 nm when the excitation wavelength was set at 280 nm (Fig. 2). The shape of the peak and the emission maximum remained the same. These observations inferred that BrNs interacts with BSA. The intrinsic fluorescence quenching can proceed via two different mechanisms. Notably, it has been demonstrated that formation of ground state complex induces static quenching and dynamic quenching results from collisional encounters. The quenching constant for static or dynamic processes can be distinguished by their different dependence on temperature^34^. In the dynamic quenching phenomenon the rise of temperature results in a faster diffusion rate of the quencher which leads to collisions at a higher rate along with an increase in quenching constant. In static process, the rise in temperature slows down the formation of complex and reduces the quenching constant.

### Quenching mechanism analysis

To assess the binding mechanism of the interaction of BrNs with BSA, fluorescence quenching study was performed at three different temperatures 288 K, 298 K and 398 K and evaluated using the Stern-Volmer equation^35^.

$$\mathrm{Stern} \mbox{-} \mathrm{Volmer\; equation}:\frac{Fo}{F}=1+{{\rm{K}}}_{{\rm{s}}{\rm{v}}}\,\times \,[{\rm{Q}}]=1+{\rm{K}}{\rm{q}}\,\times \,{\rm{t}}{\rm{o}}\,\times \,[{\rm{Q}}]$$

Here F_o_ is the fluorescence intensity prior to additions of BrNs and F represents the fluorescence intensity after the additions of BrNs. K_sv_ showing the Stern-Volmer quenching constant and (Q) showing the concentration of quencher (BrNs). The Stern-Volmer quenching constants at three different temperatures are shown in the Table 1. K_sv_ = K_q_ × τ_o,_ where K_q_ represents the quenching rate constant and τ_o_ shows the average lifetime of the biomolecules without quencher. The K_q_ values were calculated assuming the τ_o_ value to be 10^−8^s^36^. The value of quenching rate constant at all the temperatures was found to be much greater than maximum diffusion rate constant of a biomolecule^37^ i.e., 2 × 10^10^ M s^−1^ (Table 1). Therefore, it suggested that the fluorescence quenching of BSA by BrNs lead to the formation of a static complex^38^.

**Table 1:** Stern-Volmer Quenching constants of BSA-BrNs interaction at three different temperatures (288 K, 298 K, 308 K).

Temperature (K)	K_sv_ ± SD × 10^4^ (L mol^−1^)	K_q_ × 10^12^ (L mol^−1^s^−1^)	n
288	1.0531 ± 0.15	1.0531	1.13
298	0.7803 ± 0.20	0.7803	0.9673
308	0.7455 ± 0.24	0.7455	0.7805

### Binding constant and the number of binding sites

**Figure 3:**
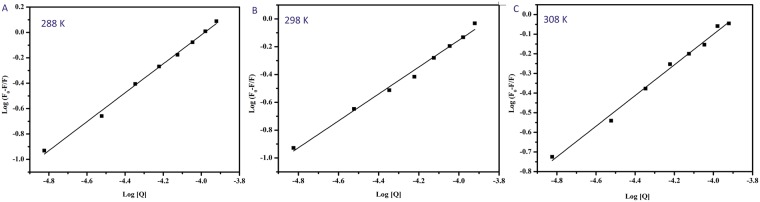
The plots of log $(\frac{Fo-F}{F})$ versus log [Q] for the BrNs-BSA complex system at (**A**) 288 K (**B**) 298 K (**C**) 308 K.

Further fluorescence data was analysed to determine the binding sites by using the modified Stern-Volemer plot^39^, as shown in the Fig. 3.

$$\mathrm{log}(\frac{Fo-F}{F})={{\rm{logK}}}_{{\rm{b}}}+{\rm{nlog}}[{\rm{Q}}]$$

Here, n is the number of binding sites for BrNs with BSA, K_b_ representing the binding constant. The plot of log $(\frac{Fo-F}{F})$ versus log [Q] was linear with the slope being equal to n and log K_b_ as the intercept. The calculated values of K_b_ were in the order of 10^3^ indicating a moderate interaction between BSA and BrNs. A noticeable decrease in the binding constant was observed with an increase in temperature because of the lower stability of BSA-BrNs complex at higher temperatures. The value of n was nearly equal to 1 for drug protein interaction at all temperatures. The decrease in the number of binding sites with increase in temperature can be attributed to the fact that with the rise in temperature, molecules are more disordered and undergo fast vibrations. This leads to higher diffusion coefficients which destabilises the BSA-BrNs complex^40^. Hence, on the basis of quenching experiments and calculated binding parameters, we concluded that the target drug BrNs produces a static complex with BSA 1:1 stoichiometry.

### Thermodynamic parameters and binding forces

**Figure 4:**
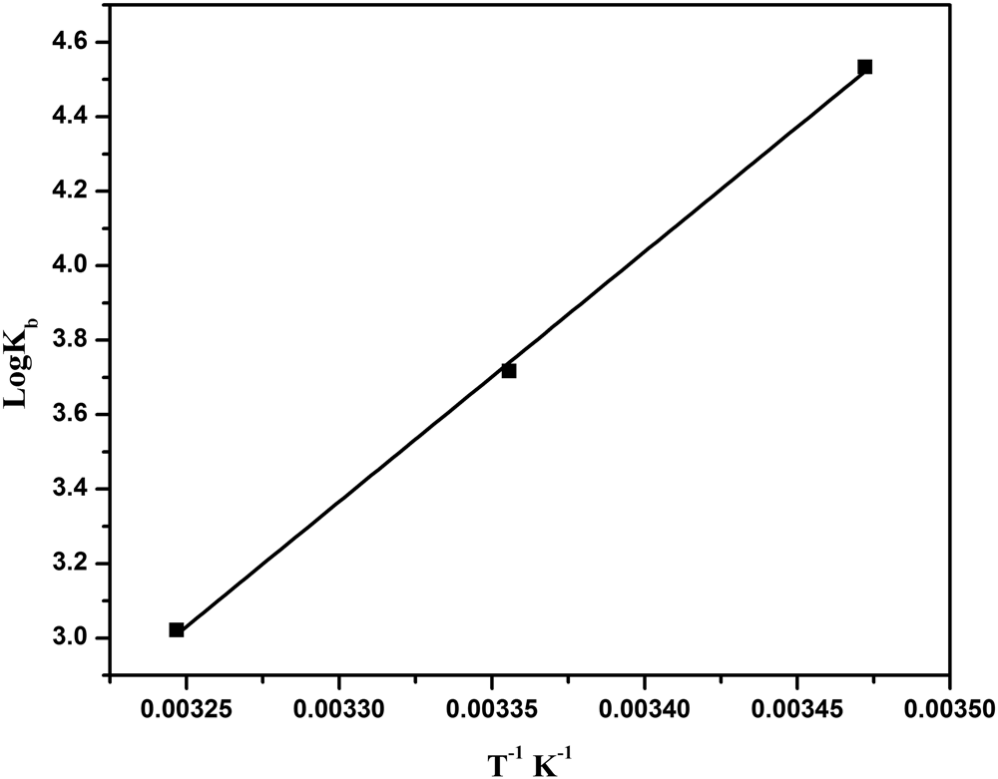
Van’t Hoff plot of log K vs 1/T for binding of BrNs with BSA.

The interaction between a biological macromolecule and a ligand generally consists of four types of forces: hydrogen bonds, van der Waals interactions, hydrophobic forces and electrostatic interactions^41^. The signs and magnitudes of the enthalpy and entropy are characteristic for each individual kind of interaction that takes place during the protein drug binding. The interacting forces of BSA-BrNs complex were identified by the thermodynamics parameters. The fluctuations in the entropy and enthalpy during the binding phenomenon were identified by van’t Hoff’s plot using the given equation (Fig. 4).

$$\mathrm{Log}\,{{\rm{K}}}_{{\rm{b}}}=-\,\frac{\Delta {\rm{H}}^\circ \,}{2.303{\rm{RT}}}+\frac{\Delta {\rm{S}}^\circ }{2.303\,R}$$

Where K_b_ is the binding constant, T is the temperature in Kelvin and R is the universal gas constant (8.314 J mol^−1^ K^−1^).

The Gibbs free energy change ΔG° was estimated according to the following relationship:

$${\rm{\Delta }}G^\circ ={\rm{\Delta }}H^\circ -T{\rm{\Delta }}S^\circ $$

The thermodynamic parameters in accordance with the binding constants at different temperature were determined and presented in Table 2. The negative value of ΔG° (Free energy) suggested the spontaneity of the interaction between BrNs with BSA. The negative values of ΔH° and ΔS° indicated that hydrogen bonds and van der Waals interactions played a potential role in binding of BrNs to BSA^42^. The ΔH° < 0 manifests that the binding process of the drug molecule was an exothermic reaction which is consistent with the decrease in the binding constant with increase in the temperature.

**Table 2:** Binding constant K_b_ and the relative thermodynamic parameters for the BSA-BrNs interaction at three variable temperatures.

Temperature (K)	K_b_ ± SD × 10^3^ (Lmol^−1^)	ΔG^0^ (kJ mol^−1^)	ΔS^0^ (J mol^−1^ K^−1^)	ΔH^0^ (kJ mol^−1^)
288	34.16 ± 0.21			
298	5.20 ± 0.16	−21.34	−359.49	−128.47
308	1.67 ± 0.31			

### UV-Visible absorption spectroscopy analysis

**Figure 5:**
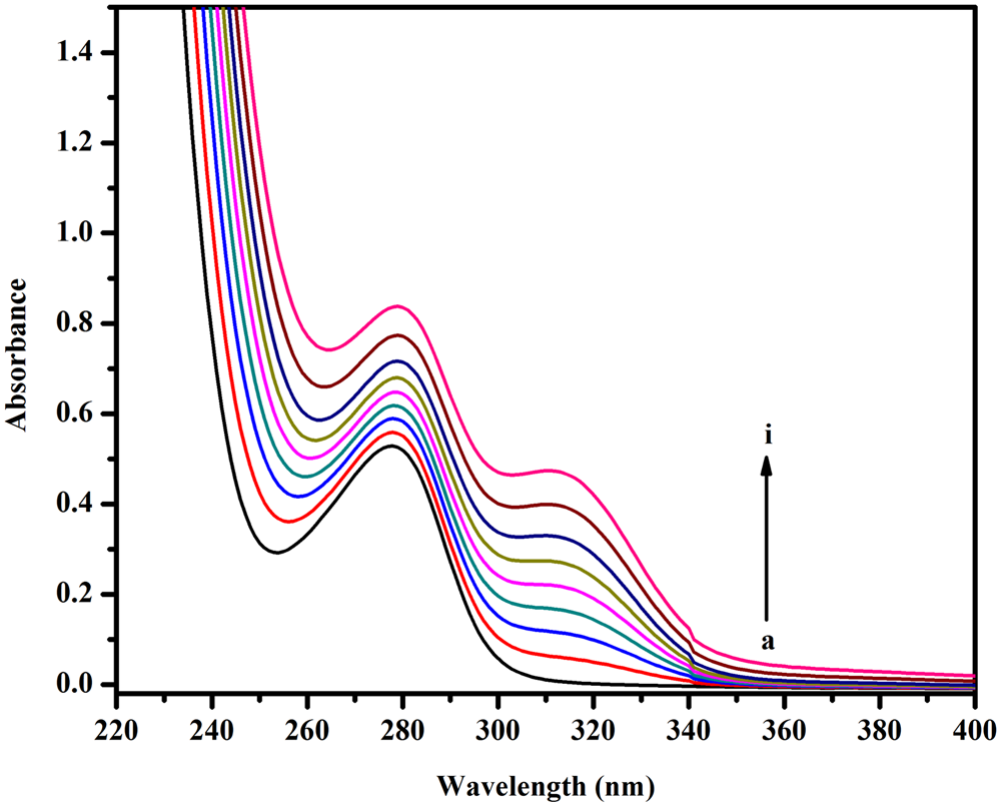
Absorbance spectra of BSA–BrNs system with increasing concentration of BrNs (**A**) 0 μM, (**B**) 15 μM, (**C**) 30 μM, (**D**) 45 μM, (**E**) 60 μM, (**F**) 75 μM, (**G**) 90 μM, (**H**) 105 μM, and (**I**)120 μM while BSA concentration was fixed at 15 μM in physiological pH 7.4 PB buffer at 298 K.

Ultraviolet absorption spectroscopy is a widely employed technique to understand protein-drug interactions and to analyse the corresponding structural changes. The absorption intensity of proteins around 280 nm belongs to the π–π* transition of the Tryptophan, Tyrosine and Phenylalanine amino acids^43^. To gain an insight into the interaction of BrNs with BSA, the spectra of the protein were examined in the absence and presence of various concentrations of the drug. An increase in the absorbance of BSA at 278 nm was observed on the successive addition of BrNs (Fig. 5). The changes in the absorption spectra are indicative of a complex formation between BSA and BrNs. These results reconfirmed that the mechanism of quenching was static because in case of dynamic quenching the absorption spectra of the protein would be unaffected by the addition of drug^44,45^.

### Analysis of the structural stability employing CD-Spectroscopy

**Figure 6:**
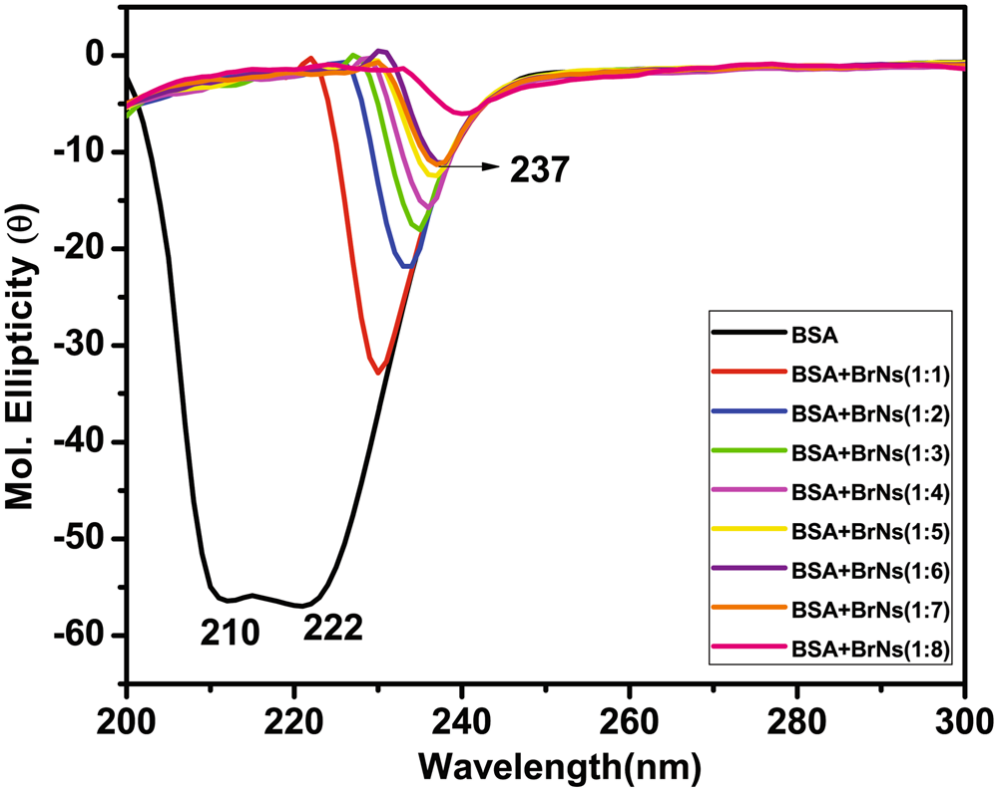
CD spectra of BSA in the absence and presence of BrNs at varied molar ratio of BSA:BrNs (1:1, 1:2, 1:3, 1:4, 1:5, 1:6, 1:7, 1:8) at pH 7.4 and 298 K temperature.

Circular dichroism is an informative method to elucidate the changes in the proteins’ secondary structure. It is also useful in understanding the interaction of ligands or drugs with proteins. The protein regions of interest were considered including the peptide bond (below 240 nm), aromatic amino acids side chains (260–320 nm) and disulfide bonds (weak broad absorption bands near 320 nm). Various intermolecular as well as intramolecular forces which involve in secondary and tertiary structure conformation of the protein can be affected by interaction with ligands, leading to further structural variations^46^. CD studies were carried out to assess the conformational changes in BSA on the successive addition of BrNs. Figure 6 depicted the CD spectra of BSA in absence as well as in the presence of BrNs in various ratios. CD spectra is manifested with two significant negative peaks centered on 209 nm and 222 nm in the UV region which revealed the n → π* shift of peptide bond, characteristic of α-helix structure^47–50^. When BrNs is used in 1:1 ratio 209 peak abruptly vanished along with the shift of 222 nm peak towards longer wavelength. Further increase in the concentration of BrNs led to the decrease in ellipticity at larger extent. The maximum wavelength shift observed was from 222 nm to 237 nm at the 1:8 ratio of BSA:BrNs. This concomitant decrease in ellipticity was indicating towards the disruption of helicity as well as destabilization of protein by BrNs.

Secondary structure estimation was performed and tabulated for BSA in absence and presence of BrNs. It is evident from the Table 3, that BSA in the absence of BrNs contains 36.8% α-helix, 26.8% turn and 36.4% random respectively. On gradual addition of BrNs, percentage of α-helix decreases abruptly along with the increment of random percentage. Randomness increases with alteration in helicity of protein. The unfolding of α-helices are predominant in the presence of BrNs in increasing concentration is very well correlated with fluorescence and UV-absorption studies. It distorts the protein structure via intercalation and disrupting its helicity. Quenching was observed in case of fluorescence studies which again support the CD studies and inference that BrNs destabilize the helix structure of BSA.

**Table 3:** Secondary structure estimation for BSA on the addition of BrNs.

Protein + Drug	α-Helix	Beta	Turn	Random
BSA	36.8%	0.0%	26.8%	36.4%
BSA + BrNs(1:1)	28.6%	0.0%	20.6%	50.8%
BSA + BrNs(1:2)	16.4%	0.0%	38.5%	45.1%
BSA + BrNs(1:3)	14.9%	0.0%	39.2%	45.9%
BSA + BrNs(1:4)	14.8%	0.0%	38.5%	46.7%
BSA + BrNs(1:5)	14.2%	0.0%	38.1%	47.7%
BSA + BrNs(1:6)	13.8%	0.0%	36.3%	49.9%
BSA + BrNs(1:7)	15.0%	0.0%	35.6%	49.4%
BSA + BrNs(1:8)	13.3%	0.0%	34.2%	52.5%

### FT-IR Spectroscopy

**Figure 7:**
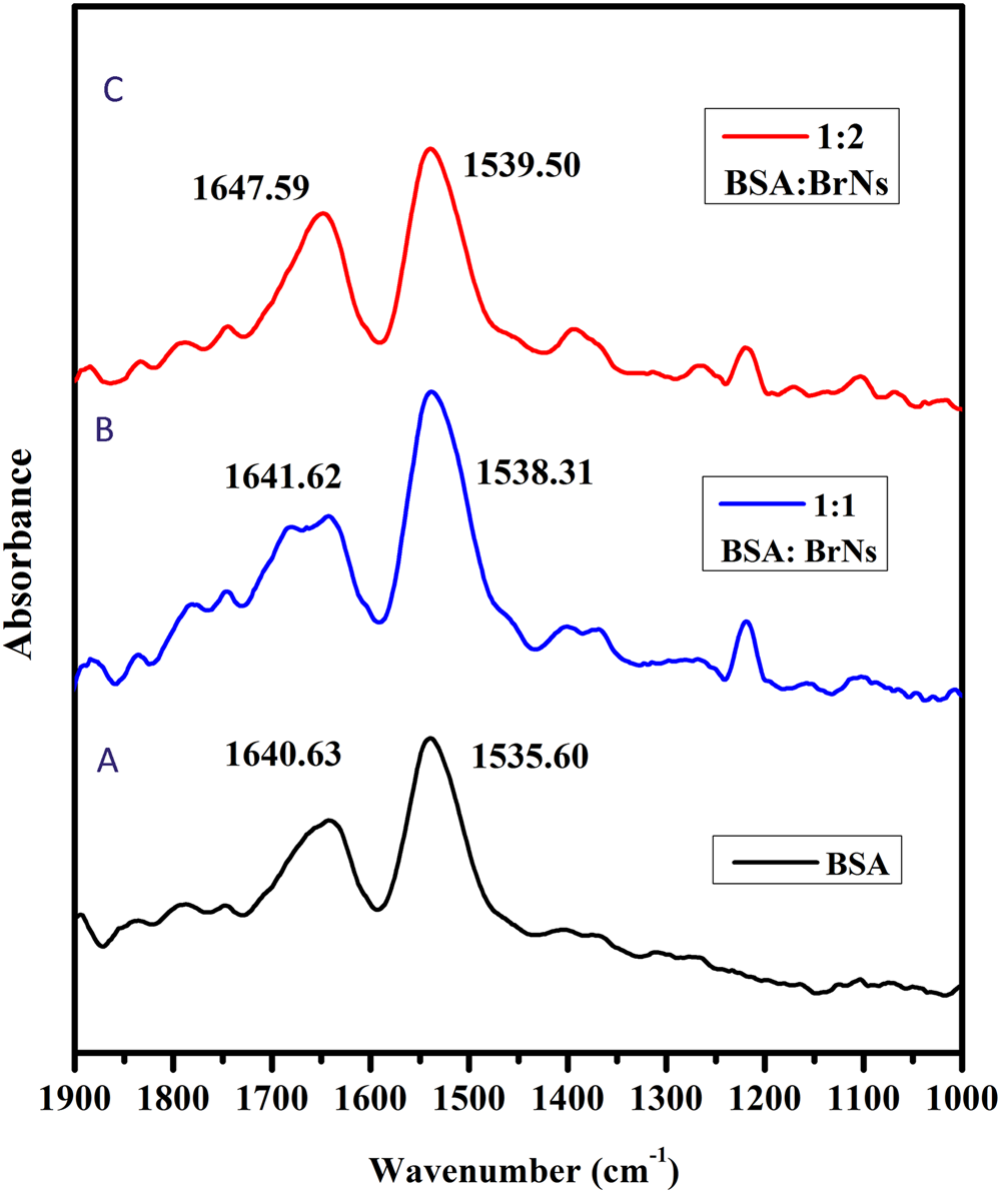
FT-IR spectra of (a) Free BSA (1.5 μM) and its drug complexes with difference spectra obtained at different drug concentrations (b) [BSA]:[BrNs] = 1:1 (c) [BSA]:[BrNs] = 1:2.

Infrared spectroscopy is a powerful tool to study the secondary structures and protein dynamics. The diverse vibrations of the peptide moiety lead to different amide bands in the infrared spectra of the proteins. The amide bands I (1600–1700 cm^−1^ region) and amide band II (around 1548 cm^−1^) are closely related to the secondary structure of the proteins^51^. However, amide I is more susceptible to the changes in protein structure than amide II. Figure 7 shows the gradual changes in the BSA with BrNs inclusions. As can be noted, the peak position shifted from 1640.63 to 1647.59 cm^−1^ in the amide I and a slight shift in amide II peak from 1535.60 to 1539.50 cm^−1^, demonstrating a change in the secondary structure of BSA after interaction with BrNs.

### Molecular docking studies

**Figure 8:**
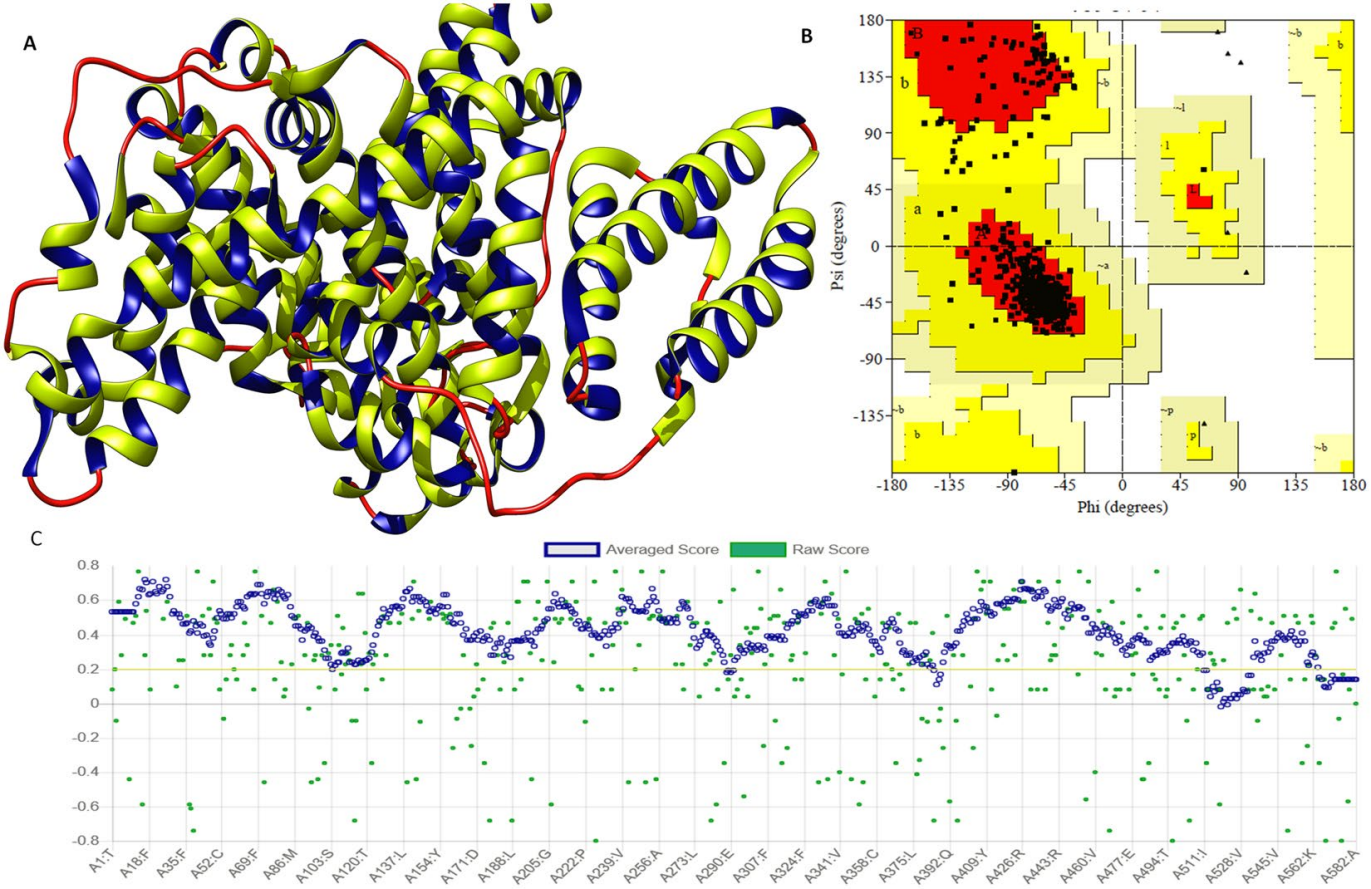
(**A**) Diagram depicting the three-dimensional structure of BSA protein in newcartoon view. Showing the alpha helices in green color with its interior in blue and random coils in red color. (**B**) Ramachandran plot of BSA protein showing 93.1% residues of structure are in the favored region, and 6.6% residues are in additionally allowed region. (**C**) Diagram depicting, BSA structure has very similar structural conformations with the native protein structures of similar size.

The 3D crystal structure of BSA was retrieved from the protein data bank (PDB ID: 4OR0). Among the various crystal structures available for BSA, 4OR0 has been crystallised at a very high resolution (2.58 Å). This structure is available as a complex with a drug and found to be a good candidate to carry out molecular docking studies. Chain-A of BSA structure was selected for molecular docking study since BSA exists as a homodimer with two chains. BSA structural properties were evaluated by Ramachandran plot analysis using the Saves server. Ramachandran plot for BSA showed that the 93.1% residues are in the most favored region and 6.6% are lying under the additionally allowed region with no residues in the outlier region. Moreover, Verify3D server also confirmed the structural quality of BSA protein with more than 80% amino acids consisting of 3D-1D profiles of native protein structures of similar size (Fig. 8).

**Figure 9:**
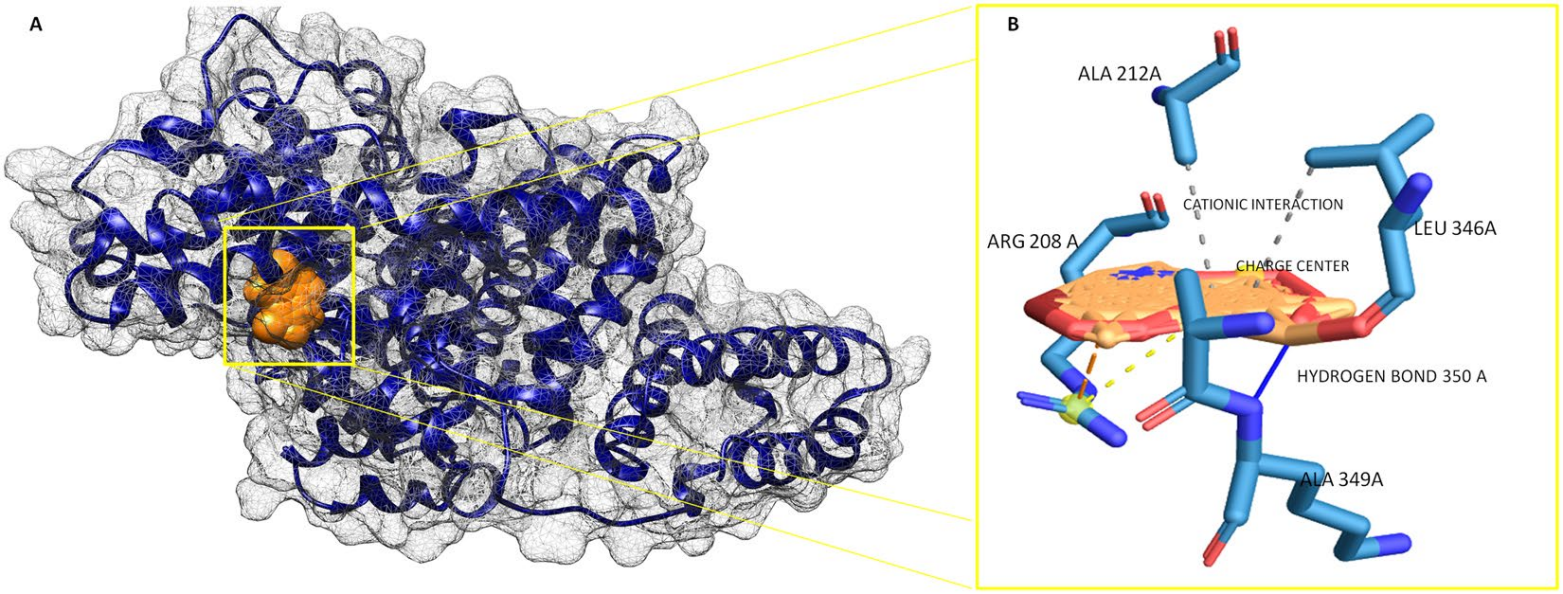
(**A**) The surface view of molecular interactions of BrNs (Orange color) to the binding groove of BSA protein (Blue color) **(B)** At the right side, enlarged view of interaction of BrNs to BSA with involved molecular interactions; hydrogen bond (Dark blue color) and hydrophobic interactions (Sky blue color).

Prior to molecular docking, water molecules were removed and hydrogen atoms were added to stabilize the BSA structure. Molecular docking analyses showed that BrNs has strong interactions with binding score of −8.08 kcal/mol using swissdock, and −231.64 kJ/mol using Hex 8.0 on the basis of shape and electrostatics parameters. The experimentally derived value for binding energy was −5.1 kcal/mol at 298 K and is closely related to the theoretically derived value^52,53^. BrNs was found to have strong binding to alpha helices domain with hydrophobic interactions at Arg208, Ala212, Ala246, Leu346 and Ala349 residues and one hydrogen bond of bond length 3.09 angstroms with Lys350 residue and pi-cation interaction with Arg208 of distance 4.15 angstroms of BSA protein (Fig. 9). The molecular docking results have successfully validated the experimental data in terms of molecular interaction and mechanism. As per thermodynamics analyses, hydrogen bonding and van der waal forces were expected, which was confirmed by *in silico* results.

### Molecular dynamics simulation study

**Figure 10:**
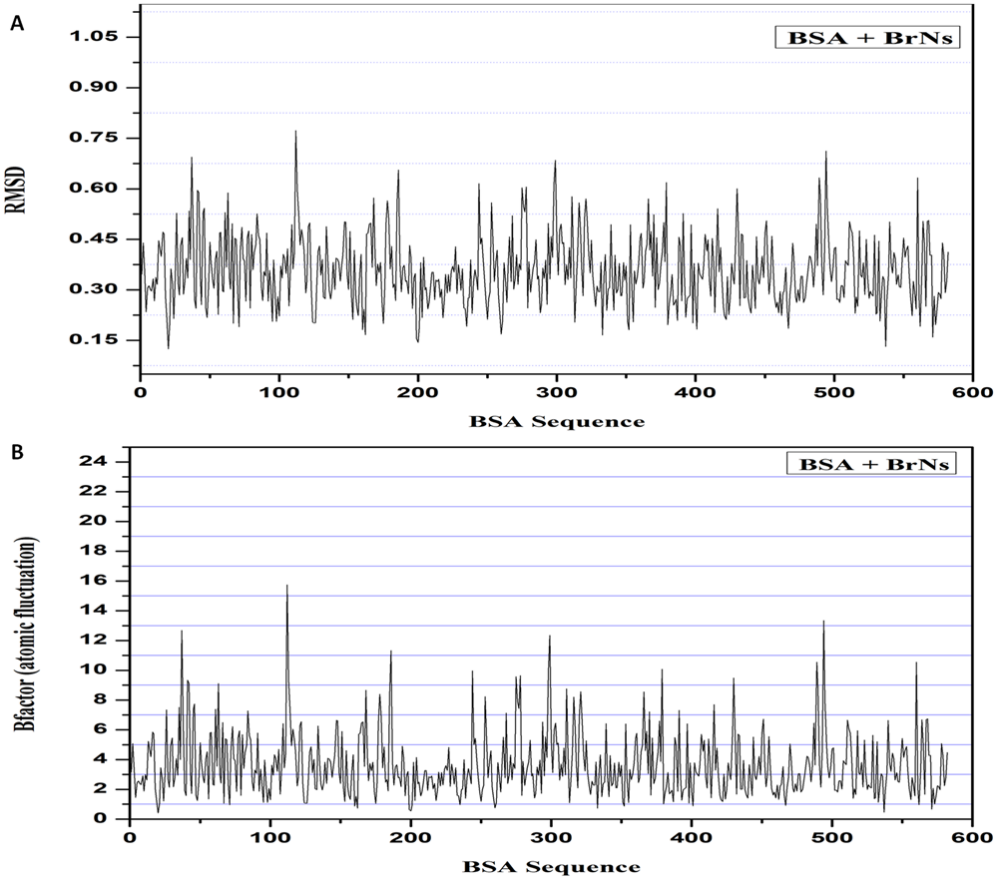
Molecular dynamics simulation plot of the BrNs and BSA protein. (**A**) The plot depicts the very less deviation RMSD structural deviation per residue of the complex. (**B**) Atomic fluctuations per residue of target protein plot of the complex with less deviation. Both plots suggest the minimal structural deviation and high stability of the interacting complex.

Molecular dynamics study of BrNs interactions with BSA was performed for 100 ps simulation run using the MDweb simulation. Multiple frames of docked complex fluctuations and structural deviations were determined. MDWeb Resulted in trajectory file of the interacting complex and which showed the minimal deviation per residue of BSA protein within the range of 0.13 to 0.78 Å in RMSD plot analysis, which suggests the stable binding of BrNs with the target BSA protein (Fig. 10). RMSF plot was also studied and showed the stable interaction of BrNs with BSA with low atomic fluctuations within a range of 1–13 Å of the interacting complex (Fig. 10). These molecular docking and simulation studies suggested the stable interaction BrNs with BSA.

## Material and Methods

### Reagents

BSA was procured from the Sisco Research Laboratories (SRL), India. BrNs was synthesized using previously reported methods. All the other chemicals used during experimentation were of analytical grade.

### Apparatus

The spectrophotometric and spectrofluorometric experiments were carried out on Cary Varian 300 Conc and Cary Eclipse, Varian, respectively. Jasco-815 CD spectropolarimeter was used to record the CD spectra. A Shimadzu II FT-IR spectrometer (USA) coupled with the Lab Solutions software was used to obtain FT-IR spectra.

### Sample Preparation

Stock solution of BrNs of 10 mM concentration was prepared in dimethylformamide (DMF). The BSA stock solution of 15 μM was prepared in 10 mM Phosphate Buffer (pH 7.4). The resulting solutions were stored in the refrigerator (2–6 ^°^C).

### Methods

#### Steady State Fluorescence Spectroscopy

The titration of the BrNs (0–120 μM) to 15 μM BSA solution was carried out. The fluorescence emission spectra of BSA were recorded at three temperatures (288, 298 and 308 K) upon excitation at 280 nm for each case. The spectra were obtained in the range of 300–500 nm with a scan speed of 600 nm min^−1^. The bandwidth for both excitation and emission were set at 5 nm.

Inner Filter Effect. The inner filter effect arises by absorption by the individual compounds in the ultraviolet region of excitation of emission wavelength and slows down the intensity of fluorescence^54,55^. The following equation was employed to correct the inner filter effect.

$${F}_{corr}={F}_{obs}{\rm{X}}\,{e}^{(Aex+Aem)/2}$$

Here F_corr_ represents the corrected fluorescence intensity and F_obs_ represents the observed fluorescence intensity. A_ex_ and A_em_ show the sum of BrNs absorbance at the excitation and emission wavelength, respectively.

#### UV- Visible Absorption Spectroscopy

UV-Visible spectra of BSA were recorded in the presence and absence of BrNs in the range of 200–400 nm. The concentration of protein was fixed at 15 μM while the BrNs concentration was varied from 0–120 μM.

#### Circular Dichroic (CD) measurements

Circular dichroism was used to map the conformational alteration in the presence of BrNs by measuring CD on a J-815 CD spectropolarimeter using a quartz cuvette of path length 0.1 cm at 1 nm data pitch intervals. All spectra were recorded in the wavelength range of 200–300 nm. The spectropolarimeter is continuously fluxed with nitrogen to absorb moisture before starting the instrument and during the experiment. The spectra were collected at a scan speed of 100 nm/min with a response time of 1 sec at 298 K temperature. The baseline correction was done for every spectrum. The average of the three accumulated plots was taken for the final spectra. The molar ellipticity [θ] is calculated from the observed ellipticity θ as

$$[\theta ]=100(\frac{\theta }{C{\rm{x}}l})$$

These experiments were performed for the $\frac{[BSA]}{[BrNos]}$ molar ratio in 1:1, 1:2, 1:3, 1:4, 1:5, 1:6, 1:7, 1:8 Secondary structure estimation is done by JASCO software Yang.jwr.

### FT-IR Spectroscopy

The FT-IR spectra of the phosphate buffer, BSA solution, BSA: BrNs solution and BrNs solution were recorded in the range 1000–1900 cm^−1^ in identical conditions. The spectrum of free BSA was obtained by digitally subtracting absorbance of the buffer from spectrum of BSA solution. Similarly, to obtain the spectrum of BSA after interaction, the absorbance of BrNs solution was subtracted from the BSA: BrNs solution.

### Molecular Docking

Molecular interactions analyses were performed employing the molecular docking of BrNs with BSA. Molecular docking was performed using the SwissDock molecular modeling suite^56^. Swiss dock worked on EA DSS engine and generated the different interaction modes to the binding region of protein along with analyzing the charm energy. Furthermore, we have also performed the molecular docking with Hex 8.0 to validate the interaction with fast fourier transformation algorithm (FTT)^57^. Hex works on using the FTT to generate the interacting complex with the lowest energy of protein and ligand.

The 3D structure of BSA was taken from the protein data bank (http://www.rcsb.org). BSA structure was prepared prior to perform molecular docking. The 3D structure was checked for its stereochemical parameters using the Ramachandran plot and with Verify3D^58,59^. BrNs was sketched using the ChemDraw and saved in the requisite format. Docked complex of BrNs and BSA were analyzed for involved molecular interaction involving the hydrophobic interaction and hydrogen bonds. Molecular modeling and interaction were studied and visualized using the Chimera.

### Molecular dynamics simulation analysis of BrNs-BSA complex

Molecular dynamics simulation studies were performed of the docked complex to evaluate the stable binding of the BrNs to BSA. Molecular dynamics simulation analyses were executed using the MDWeb program^60^. MDWeb performs the simulations using the NAMD full MD set up to scrutinize the molecular interactions trajectory. Molecular dynamics simulation process involved the drug-BSA complex structure cleaning, side chains fixing, addition of solvent box and followed by the energy minimization and equilibration of complex structure by heating the solvent at 300 Kelvin. Resulting dry trajectory was analyzed to study the RMSD and RMSF values to determine the stability and flexibility of complex.

## Conclusion

Bromo-Noscapine is a potential compound against various cancers in comparison to parent compound noscapine. It is important to understand the pharmacokinetic and pharmacodynamic properties essential for its development as an anti-cancer agent. The binding of BrNs to BSA structure has been studied systematically using the Fluorescence, FT-IR, UV, CD spectroscopy. The fluorescence results confirmed the static quenching phenomenon of BSA by BrNs. The molecular dynamics simulation studies suggested the hydrogen bond, hydrophobic and van der Waal forces are majorly responsible for drug-protein interaction and effectively prove the binding of BrNs to BSA. The potential approaches including the FT-IR, CD and UV experimental results showed that the BrNs interacts with BSA, leading to the loss of α-helix structure and the associated conformational changes in the secondary structures. Molecular docking analysis concluded the strong interaction of BrNs with BSA and results were validated with two different docking algorithms. In addition, molecular dynamics simulation studies also confirmed the stable binding of the BrNs with BSA through its persistent long simulation run with minimal fluctuations in RMSD and RMSF values. In conclusion, this study paves way to understand the binding mechanism of BrNs with blood protein BSA and helps in moving a step closer to clinical study.
